# Catalpol promotes the osteogenic differentiation of bone marrow mesenchymal stem cells via the Wnt/β-catenin pathway

**DOI:** 10.1186/s13287-019-1143-y

**Published:** 2019-01-22

**Authors:** Yu Zhu, Yanmao Wang, Yachao Jia, Jia Xu, Yimin Chai

**Affiliations:** Department of Orthopedic Surgery, Shanghai Jiao Tong University Affiliated Sixth People’s Hospital, Yishan Rd 600, Shanghai, 200233 People’s Republic of China

**Keywords:** Catalpol, Bone marrow mesenchymal stem cells, Wnt/β-catenin signalling, Bone regeneration

## Abstract

**Background:**

Rehmanniae Radix is a traditional herbal medicine in East Asia that has been widely used to treat patients with osteoporosis. However, the effect of catalpol, the primary active principle component of Rehmanniae Radix, on the function of bone marrow mesenchymal stem cells (BMSCs) and the underlying molecular mechanisms associated with its activity remain poorly understood.

**Methods:**

The effect of catalpol on the proliferation of BMSCs was evaluated using a Cell Counting Kit-8 assay. Alkaline phosphatase (ALP) staining, ALP activity and Alizarin Red staining were performed to elucidate the effect of catalpol on the osteogenesis of BMSCs. qRT-PCR, Western blotting and immunofluorescence were performed to evaluate the expression of osteo-specific markers and the Wnt/β-catenin signalling-related genes and proteins. Moreover, a rat critical-sized calvarial defect model and a rat ovariectomy model were used to assess the effect of catalpol on bone regeneration in vivo.

**Results:**

Catalpol significantly enhanced osteoblast-specific gene expression, alkaline phosphatase activity and calcium deposition in BMSCs in vitro. This phenomenon was accompanied by an upregulation of Wnt/β-catenin signalling. In addition, the enhanced osteogenesis due to catalpol treatment was partially reversed by a Wnt/β-catenin antagonist. Furthermore, catalpol increased the bone healing capacity of BMSCs in a rat critical-sized calvarial defect model and attenuated bone loss in a rat ovariectomy model.

**Conclusions:**

These data suggest that catalpol enhances the osteogenic differentiation of BMSCs, partly via activation of the Wnt/β-catenin pathway. Catalpol may provide a new strategy for bone tissue engineering and can be a potential agent for the treatment of postmenopausal osteoporosis.

**Electronic supplementary material:**

The online version of this article (10.1186/s13287-019-1143-y) contains supplementary material, which is available to authorized users.

## Background

Osteoporosis is a progressive systemic skeletal disease characterized by low bone mass, microarchitectural deterioration of the skeleton and increased bone porosity, which causes a predisposition to fractures. Osteoporosis is a major disease that affects over 200 million individuals worldwide, and an estimated 9 million new osteoporotic fractures occur every year [1, 2].

Mechanistically, osteoporosis is most often caused by an increase in bone resorption that is not sufficiently compensated for by a corresponding increase in bone formation [3]. Thus, the current clinical treatment for osteoporosis falls into two categories: antiresorptive agents that inhibit osteoclast function and anabolic agents that induce osteoblastic bone formation [4]. Currently, most pharmacological studies on osteoporosis have concentrated on bone resorption. However, antiresorptive therapy can prevent the loss of skeletal structure but cannot restore bone mass or architecture [1], and antiresorptive drugs such as bisphosphonates may lead to suppressed bone turnover after a long-term use [5, 6]. Moreover, the use of parathyroid hormone, an FDA-approved anabolic agent, is limited because it is expensive and difficult to administer [7, 8]. Thus, there is an urgent need to find a new and effective anabolic agent to treat osteoporosis.

Rehmanniae Radix, a traditional herbal medicine in East Asia, has been widely used to treat patients with osteoporosis, although its active component and mechanism of action are not fully understood [9, 10]. Catalpol is the primary active principle component of Rehmanniae Radix, which has many biological activities, including antioxidative [11], anti-inflammatory [12] and anti-ischaemic effects [13]. However, the effect of catalpol on bone formation has yet to be elucidated.

Bone formation requires an increase in the number of osteoblasts, which are derived from bone marrow mesenchymal stem cells (BMSCs). Numerous factors have been implicated in regulating BMSC differentiation towards osteoblasts, among which the Wnt/β-catenin pathway is crucial [1, 14, 15]. The binding of Wnt ligands to the Frizzled and LRP5/6 receptors leads to an inhibition of GSK-3β activity and the stabilization of β-catenin. β-Catenin then translocates to the nucleus, where it associates with the N-termini of DNA-binding proteins of the Tcf/Lef family to regulate the transcription of osteoblastogenesis-related target genes, such as Runx2 and Osterix [1]. The results of various studies have shown that the addition of agonists or the inhibition of natural antagonists of the Wnt/β-catenin pathway can increase systemic and focal bone formation [16], making this pathway an attractive target to promote bone regeneration.

In this study, we observed that catalpol enhanced the osteogenic differentiation of rat BMSCs, partly via the activation of the Wnt/β-catenin signalling pathway. Moreover, we used two animal models to assess the effect of catalpol on bone regeneration. We observed that catalpol increased the bone healing capacity of BMSCs in a rat critical-sized calvarial defect model and attenuated bone loss in a rat ovariectomy model.

## Materials and methods

### Isolation and culture of BMSCs

The isolation and culturing of BMSCs was described in our previous studies [17, 18]. Briefly, 4-week-old Sprague-Dawley rats were euthanized and sterilized using 75% ethanol for 20 min. After obtaining the femur bones under sterile conditions, the BMSCs were flushed out with modified Eagle’s medium alpha modification (α-MEM; HyClone, PA, USA) using a 5-ml syringe fitted with a 25-gauge needle. After centrifugation, the cells were cultured in α-MEM supplemented with 10% foetal bovine serum (FBS; Gibco, New York, USA) and 1% penicillin/streptomycin (Gibco), and incubated at 37 °C with 5% CO2. The cells from passages 4–6 were used in all experiments.

### Cell viability assay

The effect of catalpol on the proliferation of BMSCs was evaluated using a Cell Counting Kit-8 (CCK8; Dojindo, Kyushu Island, Japan) as described in our previous study [19]. Briefly, BMSCs were seeded into 96-well plates at 2000 cells/well with various concentrations of catalpol (10, 50 and 250 μM) in growth medium for 5 days. Cell proliferation curves were generated using a microplate reader, with the absorbance read at a wavelength of 450 nm.

### In situ senescence-associated β-galactosidase (SA-β-Gal) assay

The effect of catalpol on the senescence of BMSCs was evaluated according to a previously published study [20]. Briefly, the BMSCs were cultured in six-well plates with various concentrations of catalpol (10, 50 and 250 μM) in growth medium for 2 days. The SA-β-Gal assay was performed using a β-Galactosidase Staining Kit (Solarbio Life Sciences, Beijing, China). The percentage of SA-β-Gal-positive cells was calculated in five randomly selected fields under a light microscope.

### Osteogenic differentiation protocol

The osteogenic differentiation protocol was described in our previous studies [17, 18]. Briefly, the BMSCs were cultured in growth medium in 24-well plates at a density of 1 × 10^4^ cells/cm^2^. When over 80% confluence was reached, the medium was replaced with osteogenic induction medium (OIM), which consisted of growth medium supplemented with 1 nM dexamethasone, 50 μM l-ascorbic acid-2-phosphate and 20 mM β-glycerophosphate (Cyagen Biosciences, Guangzhou, China). Catalpol (10, 50 or 250 μM) was added to OIM in the experimental group, while the control group was cultured in OIM without catalpol. In accordance with a previous study [21], to examine the involvement of WNT/β-catenin signalling, OIM was supplemented with 50 μM catalpol in the presence or absence of 0.1 μg/ml Dickkopf-related protein 1 (DKK1; Peprotech, CT, USA), an antagonist of the WNT/β-catenin pathway.

### Adipogenic and chondrogenic differentiation protocol

For chondrogenic differentiation, the BMSCs were induced using an OriCell Mesenchymal Stem Cell Chondrogenic Differentiation Kit (Cyagen Biosciences) following the manufacturer’s instructions. Briefly, the cells were cultured in growth medium in six-well plates at a density of 2 × 10^4^ cells/cm^2^. Once 80% confluency was reached, the medium was replaced with chondrogenic induction medium supplemented with 0, 10, 50 or 250 μM catalpol. The cells were collected on day 14 of the chondrogenic induction.

The BMSCs were induced to differentiate adipogenically using an OriCell Mesenchymal Stem Cell Adipogenic Differentiation Kit (Cyagen Biosciences) following the manufacturer’s instructions. Briefly, the cells were cultured in growth medium in six-well plates at a density of 2 × 10^4^ cells/cm^2^. Once 100% confluency was reached, the medium was replaced with adipogenic induction medium supplemented with 0, 10, 50 or 250 μM catalpol. The cells were collected on day 8 of the adipogenic induction.

### ALP staining and measurement of ALP activity

ALP staining and ALP activity assays were performed using BMSCs that had been cultured in OIM for 3 days. For ALP staining, BMSCs were washed with phosphate-buffered saline (PBS) and fixed with 4% paraformaldehyde for 20 min. Next, the cells were washed three times with PBS and stained using a BCIP/NBT ALP Colour Development Kit (Beyotime, Shanghai, China) following the manufacturer’s instructions. For measurements of ALP activity, BMSCs were lysed in radioimmunoprecipitation assay (RIPA) lysis buffer (Sigma-Aldrich, MO, USA). The ALP activity in the cellular fraction was measured using a microplate test kit (Nanjing Jiancheng Biotechnology Co Ltd., Jiangsu, China) following the manufacturer’s instructions, and the absorbance at 520 nm was measured using a microplate reader.

### Alizarin Red staining

Alizarin Red staining was performed to evaluate calcium deposit formation after 14 days of osteogenic induction. Cells were fixed with 4% paraformaldehyde for 20 min and then were washed three times with PBS. Next, the cells were stained with Alizarin Red (Cyagen Biosciences) for 5 min at room temperature and were subsequently washed three times with PBS. To quantify mineralization, the calcium deposition was desorbed with 10% cetylpyridinium chloride (Sigma-Aldrich), after which the solution was collected, and the OD was measured at 570 nm.

### RNA extraction and quantitative reverse transcription polymerase chain reaction (qRT-PCR)

Total RNA was extracted from the cultured BMSCs 7 days after osteogenic induction using TRIzol reagent (Ambion, New York, USA). A reverse transcription procedure was carried out using a High-Capacity RNA-to-cDNA kit (Takara Bio, Otsu, Japan). Quantitative PCR was performed on an ABI StepOnePlus System (Applied Biosystems, Warrington, UK) using SYBR Green I Master Mix (Takara Bio) following the manufacturer’s instructions. The primers were provided by BioTNT (BioTNT, Shanghai, China), the sequences of which were as follows: *Col1* forward: 5′ CATCGGTGGTACTAAC 3′, reverse: 5′ CTGGATCATATTGCACA 3′; *Alp* forward: 5′ ACCATTCCCACGTCTTCACATTT 3′, reverse: 5′ AGACATTCTCTCGTTCACCGCC 3′; osteocalcin (*Ocn*) forward: 5′ CCTCACACTCCTCGCCCTATT 3′, reverse: 5′ CCCTCCTGCTTGGACACAAA 3′; runt-related transcription factor 2 (*Runx2*) forward: 5′ ACTTCCTGTGCTCGGTGCT 3′, reverse: 5′ GACGGTTATGGTCAAGGTGAA 3′; *β-catenin* forward: 5′ CTTACGGCAATCAGGAAAGC 3′, reverse: 5′ TAGAGCAGACAGACAGCACCTT 3′; *Gsk-3β* forward: 5′ ATAGGTGACAGGCACAACGACA 3′, reverse: 5′ CGGGGTTAGGACAAAAGGTACTC 3′; and glyceraldehyde-3-phosphate dehydrogenase (*Gapdh*) forward: 5′ GGCATGGACTGTGGTCATGAG 3′, reverse: 5′ TGCACCACCAACTGTTAGC 3′. PCR was performed for 40 cycles, and the mRNA expression levels of target genes were analysed relative to the mRNA expression levels of Gapdh using the 2^−ΔΔCt^ method.

### Western blot (WB) analysis

BMSCs were collected after 7 days of osteogenic induction. The bone tissues in the defect areas of the craniums were sampled after the rats were euthanized 8 weeks after surgery. Cell or tissue samples were lysed in RIPA lysis buffer (Sigma-Aldrich) containing a proteasome inhibitor (Beyotime). For nuclear protein preparations, cells were washed twice with PBS, and the proteins in the nucleus were extracted using a Nuclear and Cytoplasmic Protein Extraction Kit (Beyotime), following the manufacturer’s protocol. Protein concentrations were determined using a BCA protein assay kit (Cell Signaling Technology, MA, USA). Next, 10 μg of protein was separated by 10% sodium dodecyl sulfate polyacrylamide gel electrophoresis and was subsequently transferred to polyvinylidene fluoride membranes (Millipore, MA, USA). The membranes were blocked in 5% non-fat milk for 2 h and then were incubated overnight with primary antibodies specific to RUNX2 (1:2500, Cell Signaling Technology), COL1 (1:5000, Abcam, MA, USA), β-catenin (1:5000, Abcam), GSK-3β (1:1000, Cell Signaling Technology), Ser9 phosphorylation GSK-3β (P-GSK-3β; 1:1000, Signalway Antibody, MD, USA), LEF1 (1:2000, Cell Signaling Technology), TCF1/7 (1:500, Cell Signaling Technology), COL2 (1:1000, Proteintech Group, IL, USA), SOX-9 (1:1000, Santa Cruz Biotechnology, TX, USA), PPARγ (1:1000, Proteintech Group), ERK1/2 (1:2000, Cell Signaling Technology), P-ERK1/2 (1:2000, Cell Signaling Technology), p38 (1:1000, Proteintech Group), P-p38 (1:1000, Cell Signaling Technology), SMAD1/5/8 (1:1000, Affinity Biosciences, OH, USA), P-SMAD1/5/8 (1:1000, Cell Signaling Technology), HISTONE (1:1000, Proteintech Group) and GAPDH (1:4000, Cell Signaling Technology). After rinsing, the membranes were incubated with horseradish peroxidase-conjugated secondary goat anti-rabbit IgG (1:5000; Proteintech Group, IL, USA) for 1 h at room temperature. After chemiluminescence, an ECL Plus Western Blotting Detection System (GE Healthcare, IL, USA) was used to visualize the target bands.

### Immunofluorescence (IF)

After cells were cultured in OIM for 7 days, COL1, RUNX2 and β-catenin were detected by IF. Briefly, BMSCs were fixed in 4% paraformaldehyde for 10 min, permeabilized with 0.25% Triton X-100 for 10 min and blocked with 1% bovine serum albumin for 30 min. After washing, the cells were incubated overnight at 4 °C with primary antibodies specific to COL1 (1:500, Abcam), RUNX2 (1:250, Cell Signaling Technology) and β-catenin (1:200, Abcam). Subsequently, the cells were incubated with Alexa-488-conjugated secondary antibody (1:800, Jackson, PA, USA) for 2 h at room temperature. Nuclei were stained with DAPI (Sigma-Aldrich), and actin filaments were labelled with TRITC Phalloidin (Yeasen Biotechnology, Shanghai, China). The fluorescence intensity was evaluated in five random fields using ImageJ 1.8.

### Critical-sized calvarial defect model

ShakeGel™ 3D hydrogel (Biomaterials USA, VA, USA), a functionalized polysaccharide-based bioactive hydrogel, was used as the scaffold to load BMSCs [22]. By being mixed with fluids containing metal ions, this hydrogel can form three-dimensional nanofibre networks that mimic the microenvironment and microstructures of tissues in supporting 3D tissue-like growth in vivo. Cells were mixed into the hydrogel following the manufacturer’s protocol. Briefly, cells were suspended in growth medium at 2 × 10^6^ cells/mL and mixed with an equal volume of the hydrogel. The cell/hydrogel composites were then applied to the calvarial bone defects in rats. For groups implanted with BMSCs treated with catalpol, the cells were precultured with 50 μM catalpol for 5 days before being mixed with the hydrogel. To ensure that the cells were exposed to catalpol in vivo, catalpol was added to the mixed hydrogel composites at a final concentration of 50 μM. In the group implanted with the BMSCs treated with catalpol + DKK1, the cells were precultured with 50 μM catalpol and 0.1 μg/ml DKK1 for 5 days before being mixed with the hydrogel. Before implantation, catalpol and DKK1 were added to the mixed hydrogel composites at final concentrations of 50 μM and 0.1 μg/ml, respectively.

All experimental procedures were approved by the Animal Research Committee of Shanghai Jiao Tong University Affiliated Sixth People’s Hospital. According to previously published studies [22, 23], 6-week-old male Sprague-Dawley rats were anaesthetized, and two 5-mm critical-sized calvarial defects were carefully made on each side of the cranium using a trephine drill, avoiding damage to the dura mater. The hydrogels or cell/hydrogel composites were then implanted into the defects. Thirty rats were randomly assigned to three groups for the following implants: (1) hydrogel alone (control group) (*n* = 10), (2) hydrogel mixed with BMSCs (BMSCs group) (*n* = 10) and (3) hydrogel mixed with BMSCs treated with catalpol (BMSCs + CA group) (*n* = 10). Eight weeks after surgery, osteogenesis and angiogenesis were evaluated.

To assess bone regeneration, the craniums of rats were harvested after they were euthanized. The craniums were assessed by microCT scanning, WB analysis and non-decalcified histological assessments. To assess angiogenesis, vascular perfusion was performed according to previous studies [24, 25]. Briefly, after the rats were anaesthetized, 10 ml of MICROFIL (Flow Tech, MA, USA) was perfused into the left ventricle. Next, the craniums were harvested, decalcified and assessed by microCT scanning. A 3D reconstruction of blood vessels was performed, and the morphometric parameters of the vessel area and vessel number in the bone defect area were calculated.

### Rat ovariectomy model

All experimental procedures were approved by the Animal Research Committee of Shanghai Jiao Tong University Affiliated Sixth People’s Hospital. According to previously published studies [4, 21], thirty 8-week-old female Sprague-Dawley rats were randomly assigned to three groups: (1) sham: sham surgery followed by PBS vehicle treatment, (2) ovariectomized (OVX): ovariectomy followed by PBS vehicle treatment and (3) OVX + CA: ovariectomy followed by catalpol treatment. Vehicle or catalpol (10 mg/kg per day) [26] was administered by intraperitoneal injection for 8 weeks after surgery. Eight weeks after surgery, the rats were sacrificed, and the femurs were collected for microCT and histological analyses.

### MicroCT analysis

The craniums and femurs of rats were scanned using a microCT scanner (Skyscan 1172, Bruker microCT, Kontich, Belgium) with the following parameters: an X-ray energy setting of 80 kVp, a current of 112 μA and an exposure time of 370 ms. For the craniums, the parameters of the new bone volume/total volume (BV/TV) and bone mineral density (BMD) of the bone defect area were recorded for analysis. For the femurs, six trabecular morphometry parameters were evaluated, namely, the BV/TV, trabecular number (Tb.N), trabecular thickness (Tb.T), BMD, trabecular separation (Tb.Sp) and structure model index (SMI) of the distal femur. In addition, two cortical parameters were quantitatively measured, namely, the cortical bone area (Ct.Ar) and cortical thickness (Ct.Th) of the mid-diaphysis. Representative three-dimensional transverse images were reconstructed from the level 2.0 mm proximal to the growth plate encompassing 1 mm of the distal metaphysis.

### Histological analysis

For the craniums, after serial dehydration, the specimens were embedded in polymethylmethacrylate. After hardening, the specimens were sectioned coronally through the central area of the defect at a thickness of 5 μm with a microtome (Leica, Hamburg, Germany). Next, van Gieson’s (VG) picrofuchsin staining and Goldner’s trichome staining were performed to evaluate new bone formation in accordance with previous studies [22, 23]. For femurs, after decalcification with 10% EDTA, the samples were embedded in paraffin and sectioned coronally at a thickness of 5 μm. Haematoxylin and eosin (H&E) staining was then performed according to previous studies [21].

### Statistical analysis

Statistical analysis was performed using SPSS 22.0 (IBM, New York, USA). Data are presented as the means ± standard deviation (SD). Considering the relatively small sample sizes, comparisons of in vitro experiments were performed using the Wilcoxon-Mann-Whitney test [27]. Comparisons of animal experiments were made using a two-tailed Student’s *t* test, with *P* < 0.05 considered significant.

## Results

### Catalpol does not affect BMSC proliferation

**Fig. 1:**
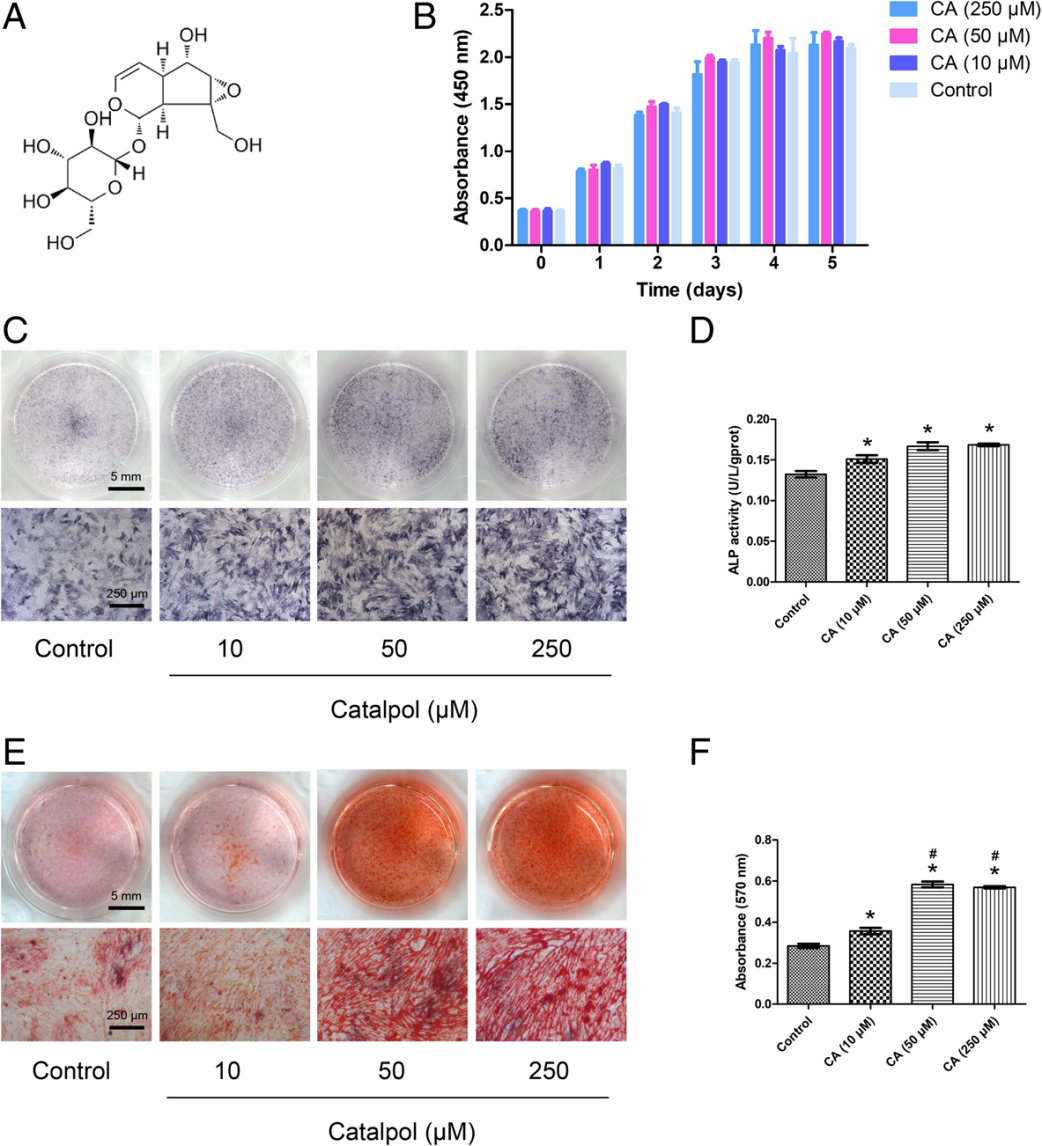
Catalpol promotes the osteogenic differentiation of BMSCs in vitro. **a** Chemical structure of catalpol. **b** Cell viability after catalpol treatment was determined using the CCK-8 assay. **c**–**f** Osteogenic differentiation was assessed by ALP staining (**c**), ALP activity assays (**d**) and Alizarin Red staining (**e**). Calcium deposition was determined by measuring optical density (**f**). The data were confirmed by three repeated tests. The data are presented as the means ± SD. CA, catalpol. *P < 0.05 compared with the control group, ^#^*P* < 0.05 compared with the 10 μM catalpol treatment group

Catalpol is a natural iridoid glycoside, which is a simple monoterpene with a glucose molecule attached (Fig. 1a). To determine whether catalpol influences the proliferation of BMSCs, a CCK8 assay was performed. Cell proliferation was not significantly affected by treatment with 10, 50 or 250 μM catalpol for 1–5 days compared to the control group (*P* > 0.05) (Fig. 1b), indicating that catalpol had no significant cytotoxic effects on BMSCs at these concentrations. In addition, catalpol had no effect on the senescence of the BMSCs at the assayed concentrations as indicated by the SA-β-Gal assay results (*P* > 0.05) (see Additional file 1).

### Catalpol enhances ALP activity and deposition of calcium

To elucidate the effect of catalpol on the osteogenesis of BMSCs in vitro, ALP staining, ALP activity assays and Alizarin Red staining were performed. ALP is an early marker of osteogenesis, and enhanced ALP staining and higher ALP activity were observed in the catalpol treatment groups compared to the control group (*P* < 0.05), while no significant differences were observed between the 10, 50 and 250 μM catalpol treatment groups (*P* > 0.05) (Fig. 1c, d). The number of calcium deposits, which was examined by Alizarin Red staining, was significantly increased after catalpol treatment (*P* < 0.05), and the mineralization was significantly enhanced in the 50 and 250 μM groups compared with that observed in the 10 μM group (*P* < 0.05). However, no significant difference in mineralization was observed between the 50 and 250 μM groups (*P* > 0.05) (Fig. 1e, f).

### Catalpol increases the expression of osteogenic differentiation-related marker genes and proteins

**Fig. 2:**
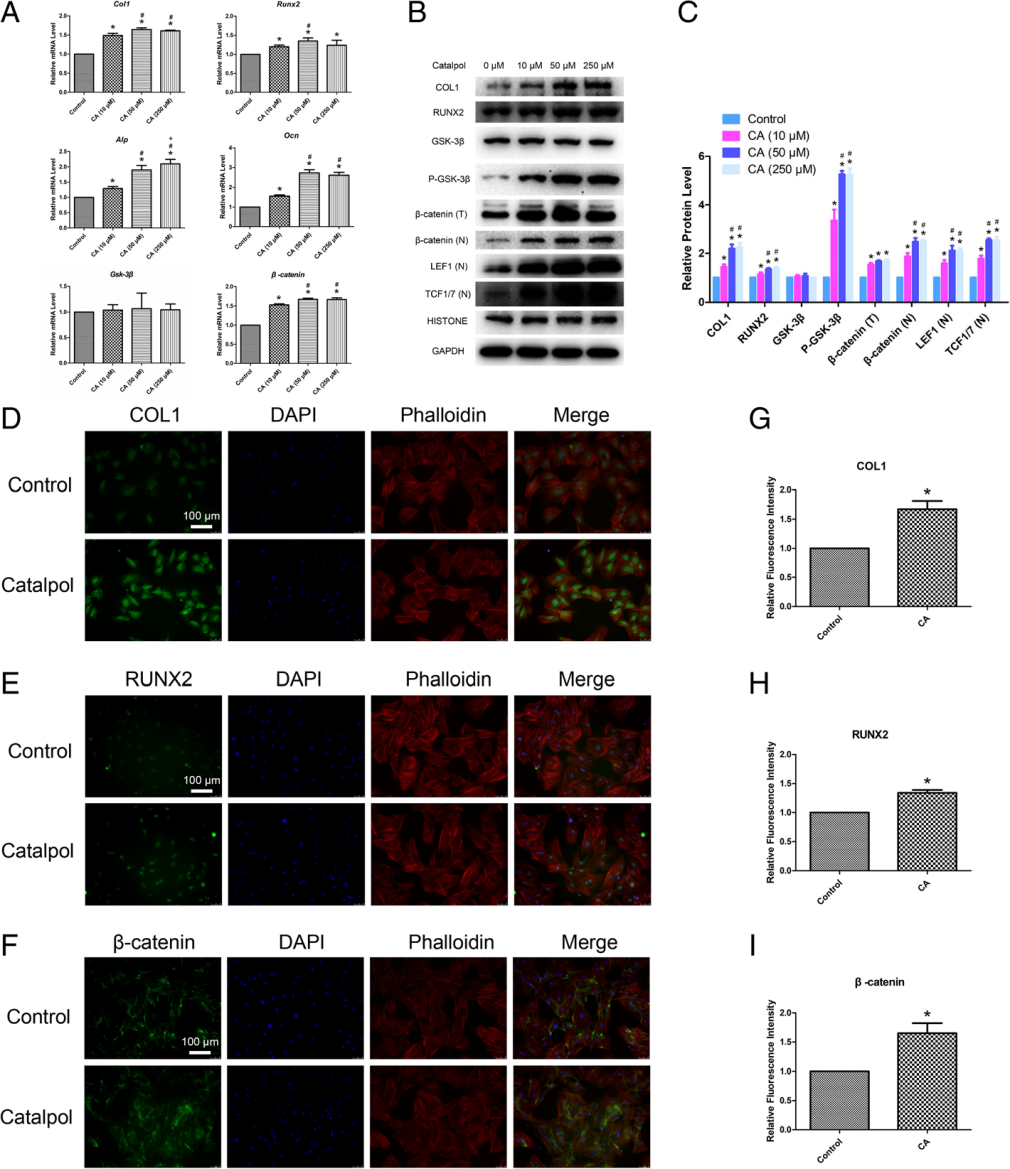
Catalpol increases the expression of osteogenic-specific genes and proteins and activates Wnt/β-catenin signalling. **a**–**c** The expression of osteogenic-specific and Wnt/β-catenin signalling-related genes and proteins were assessed by qRT-PCR (**a**) and WB (**b**, **c**) at day 7 of osteogenic differentiation. **d**–**i** The expression of osteogenic-specific proteins COL1 (**d**, **g**) and RUNX2 (**e**, **h**) and β-catenin, the key protein of Wnt/β-catenin signalling (**f**, **i**), were assessed by IF. The data were confirmed by three repeated tests. The data are presented as the means ± SD. CA, catalpol; β-catenin (T), total β-catenin; β-catenin (N), nuclear β-catenin; LEF1 (N), nuclear LEF1; TCF1/7 (N), nuclear TCF1/7. *P < 0.05 compared with the control group, ^#^*P* < 0.05 compared with the 10 μM catalpol treatment group

The expression levels of *Col1*, *Runx2*, *Alp* and *Ocn*, which are osteo-specific genes, were measured by qRT-PCR. The mRNA expression levels of *Col1*, *Runx2*, *Alp* and *Ocn* were significantly increased after catalpol treatment at day 7 (*P* < 0.05) (Fig. 2a). Furthermore, WB and IF were performed to confirm the effect of catalpol on the protein levels of COL1 and RUNX2. The results showed that higher levels of COL1 and RUNX2 protein expression were observed after catalpol treatment (*P* < 0.05) (Fig. 2b–e, g, h).

In addition, the results of the WB analysis demonstrated that the expression levels of the chondrogenic markers COL2 and SOX-9 were unaffected by the catalpol treatment (*P* > 0.05), while the adipogenic marker PPARγ was decreased after the catalpol treatment (*P* < 0.05) (see Additional file 2).

### Catalpol activates Wnt/β-catenin signalling

Wnt/β-catenin signalling has been shown to play a crucial role in the osteogenesis of BMSCs. To determine the mechanism through which catalpol affects BMSC differentiation, we investigated the expression of genes and proteins associated with the Wnt/β-catenin signalling pathway after catalpol treatment. The qRT-PCR results demonstrated that *β-catenin* expression was enhanced after catalpol treatment (*P* < 0.05), whereas *Gsk-3β* did not significantly change (*P* > 0.05) (Fig. 2a). WB analyses demonstrated that the expression levels of P-GSK-3β, total β-catenin and nuclear β-catenin were significantly enhanced after catalpol treatment (*P* < 0.05), whereas GSK-3β levels did not significantly change (*P* > 0.05). Nuclear LEF1 and TCF1/7, which are the downstream signalling molecules of Wnt/β-catenin signalling, were also upregulated (*P* < 0.05) (Fig. 2b, c). The results of IF analyses also confirmed that β-catenin expression was significantly enhanced after catalpol treatment (*P* < 0.05) (Fig. 2f, i).

In addition, the results of the WB analyses revealed that the expression levels of P-ERK1/2, P-p38 and P-SMAD1/5/8 were increased after the catalpol treatment (*P* < 0.05), indicating the involvement of the MAPK and BMP signalling pathways (see Additional file 2).

### DKK1 can partially inhibit the increased osteogenesis of BMSCs promoted by catalpol treatment

**Fig. 3:**
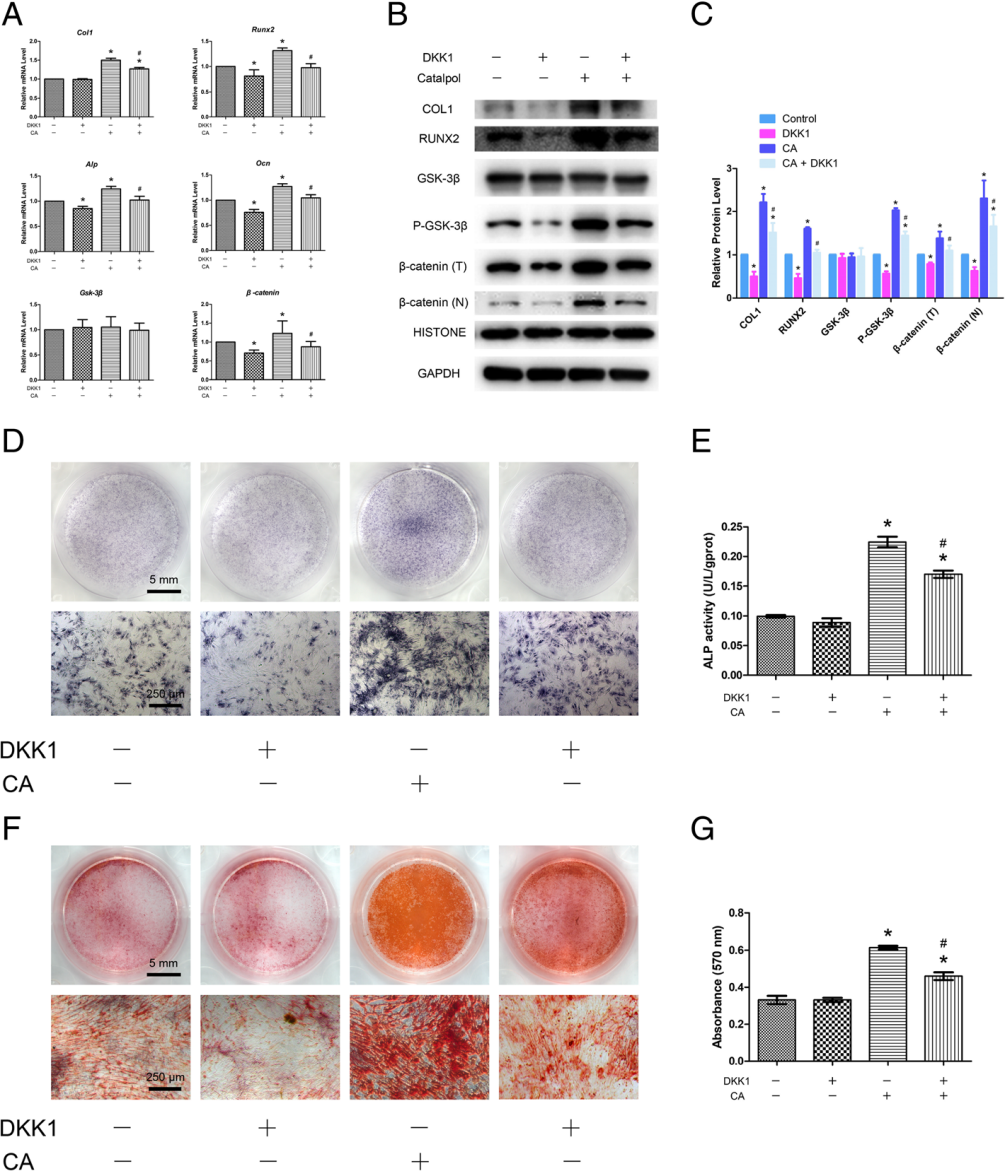
The increased osteogenesis of BMSCs caused by catalpol treatment can be partially inhibited by a Wnt/β-catenin signalling inhibitor (DKK1). **a**–**c** The expression of osteogenic-specific and Wnt/β-catenin signalling-related genes and proteins were assessed by qRT-PCR (**a**) and WB (**b**, **c**) at day 7 of osteogenic differentiation. **d**–**g** Osteogenic differentiation was assessed by ALP staining (**d**), ALP activity assays (**e**) and Alizarin Red staining (**f**). Calcium deposition was determined by optical density analysis (**g**). The data were confirmed by three repeated tests. The data are presented as the means ± SD. CA, catalpol; β-catenin (T), total β-catenin; β-catenin (N), nuclear β-catenin. *P < 0.05 compared with the control group, ^#^*P* < 0.05 compared with the catalpol treatment group

**Fig. 4:**
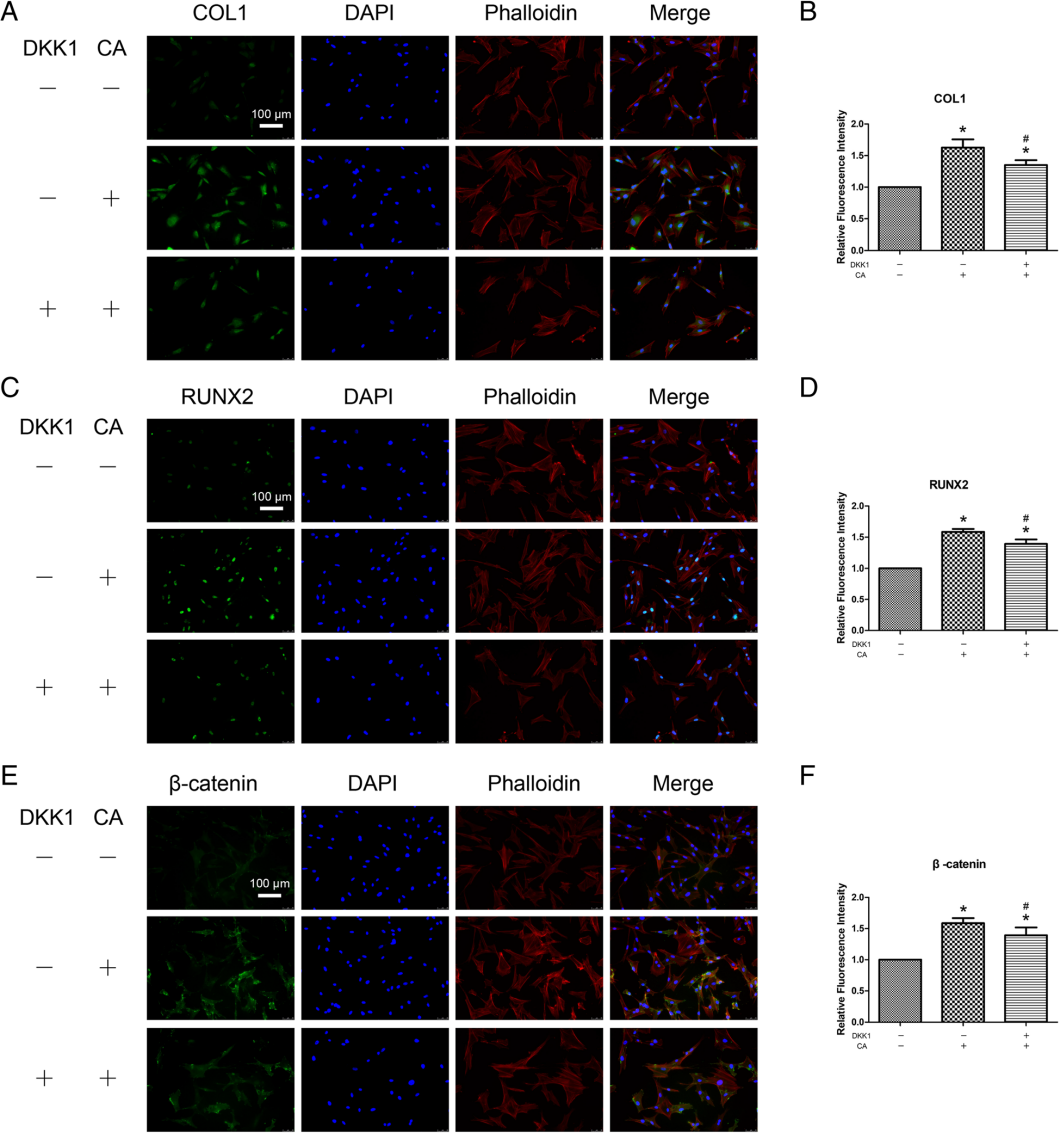
Immunofluorescence staining shows the protein levels of COL1 (**a**, **b**), RUNX2 (**c**, **d**) and β-catenin (**e**, **f**) with the administration of DKK1. The data were confirmed by three repeated tests. The data are presented as the means ± SD. CA, catalpol. *P < 0.05 compared with the control group, ^#^*P* < 0.05 compared with the catalpol treatment group

To further evaluate the involvement of Wnt/β-catenin signalling, the inhibitory effect of this pathway on osteogenesis in the catalpol treatment group was evaluated. With DKK1 treatment, the levels of P-GSK-3β and β-catenin expression in BMSCs treated with catalpol were significantly decreased compared with those observed in cells without inhibitor treatment (Figs. 3a–c and 4). Moreover, inhibition of Wnt/β-catenin signalling partially reversed the positive effects of catalpol on the expression of osteo-specific genes and proteins, as demonstrated by the qRT-PCR, WB and IF results (Figs. 3a–c, 4). In addition, ALP staining and activity assay results revealed higher ALP expression in the catalpol group than in the catalpol + DKK1 group (Fig. 3d, e), and similar calcium deposition was observed by Alizarin Red staining (Fig. 3f, g).

The dose-response of DKK1 was also evaluated. Compared with a dosage of 0.1 μg/ml, at the 0.3 μg/ml dosage, DKK1 further decreased COL1 expression and the calcium deposition of BMSCs after the catalpol treatment (see Additional file 3). In addition, as the noncanonical Wnt pathway also participates in osteogenesis [28], we assessed the effect of SP600125, an inhibitor of the Wnt/PCP pathway, to evaluate the role of the noncanonical Wnt pathway in the effect of catalpol. The results of the WB analysis demonstrated that the inhibition of the Wnt/PCP pathway also partially reversed the upregulation of RUNX2 and COL1 following the catalpol treatment. Furthermore, compared with DKK1 alone, the DKK1 + SP600125 group exhibited increased inhibition of COL1 expression (see Additional file 4).

### Catalpol increases the bone healing capacity of BMSCs in a rat critical-sized calvarial defect model

**Fig. 5:**
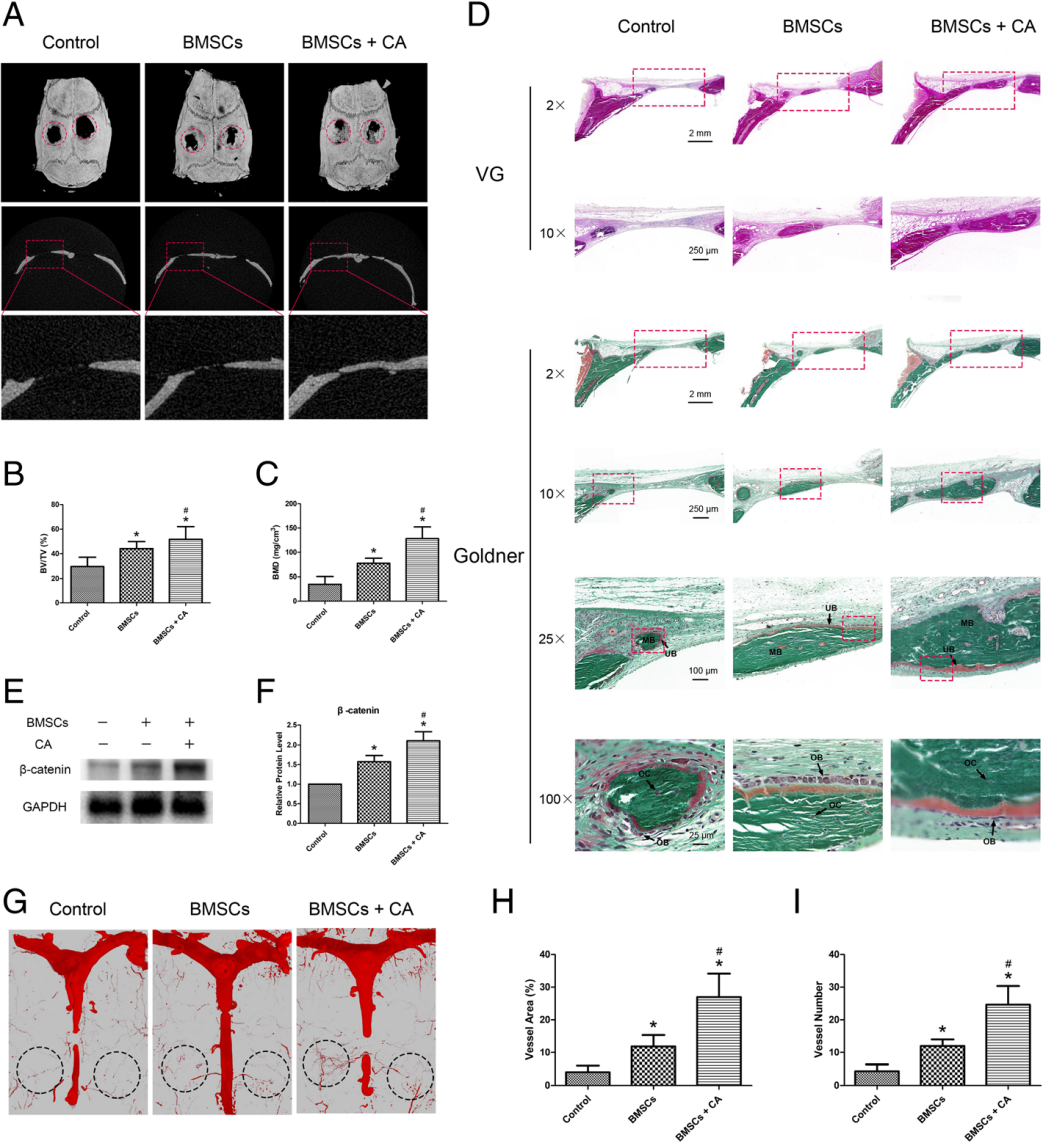
Catalpol increases the bone healing capacity of BMSCs in a rat critical-sized calvarial defect model. **a** MicroCT 3D reconstruction and coronal images of the defect area at 8 weeks after surgery in each group. **b**, **c** MicroCT analyses of bone volume/total volume (BV/TV) and bone mineral density (BMD). **d** Histological evaluation of the defect area by van Gieson’s picrofuchsin staining and Goldner’s trichome staining at 8 weeks after surgery in each group. Goldner’s trichome staining showed green-stained mineralized bone (MB) and red-stained unmineralized bone (UB). Osteoblasts (OB) were located on the surfaces of the UB, and osteocytes (OT) were also observed. **e**, **f** WB analysis of β-catenin expression in the defect areas at 8 weeks after surgery in each group. **g** 3D reconstruction images of blood vessels examined by microCT scanning. **h**, **i** Quantification of microCT images of the vessel area and vessel number in the defect area. The data were confirmed by three repeated tests. The data are presented as the means ± SD. VG, van Gieson’s picrofuchsin staining; Goldner, Goldner’s trichome staining; CA, catalpol. *P < 0.05 compared with the control group, ^#^*P* < 0.05 compared with the BMSC group

Eight weeks after surgery, the newly formed bone in the defect area of the craniums was assessed using microCT scanning. The 3D reconstruction and coronal images rarely showed newly formed bone in the control group. However, the BMSC group showed an increase in bone formation. Additionally, the BMSCs + CA group showed a larger extent of bone regeneration, which occupied almost the entire defect area (Fig. 5a). This trend was confirmed by the results of the BV/TV (Fig. 5b) and BMD (Fig. 5c) quantitative analyses.

Consistent with the above findings, the histological results of VG and Goldner’s staining demonstrated that the BMSCs + CA group showed increased mineralized bone tissues within the defect areas (Fig. 5d). In addition, the results of the WB analysis demonstrated that the level of β-catenin was enhanced in the bone defect areas after catalpol treatment (Fig. 5e, f). The 3D reconstruction images of the blood vessels demonstrated that compared to the control or BMSCs groups, the BMSCs + CA group showed markedly enhanced angiogenesis in the bone defect area (Fig. 5g). The vessel area and vessel number quantification results verified this trend (Fig. 5h, i).

In addition, the inhibitory effect of Wnt/β-catenin signalling was evaluated. As shown in Additional file 5, the microCT images and quantitative analysis revealed that the BMSCs + CA + DKK1 group showed less newly formed bone than the BMSCs + CA group.

### Catalpol attenuates the bone loss induced by ovariectomy

**Fig. 6:**
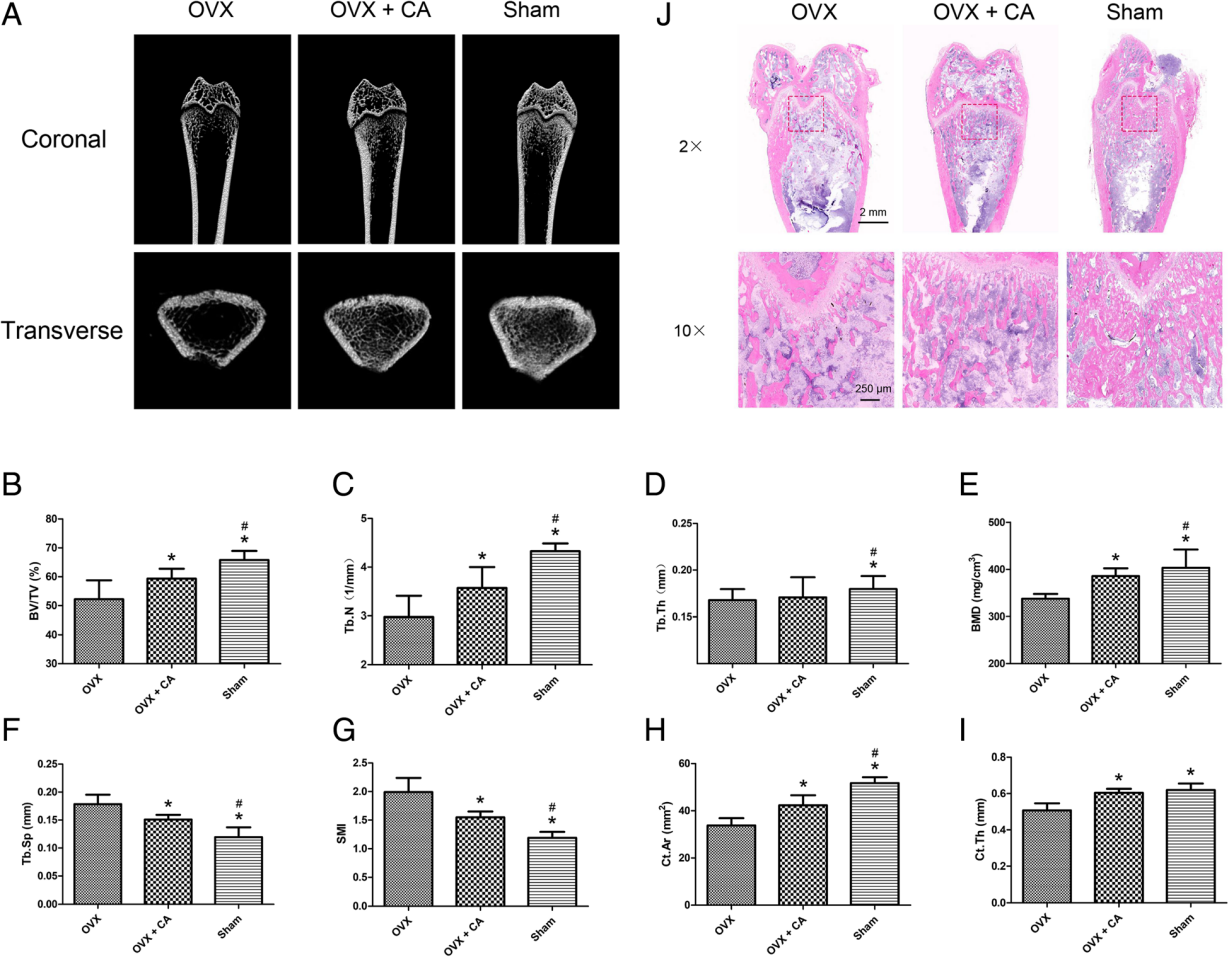
Catalpol attenuated the bone loss induced by ovariectomy. **a** 3D reconstruction of the coronal and transverse images of the distal femur at 8 weeks after surgery in each group. **b**–**i** MicroCT analyses of bone volume/total volume (BV/TV), trabecular number (Tb.N), trabecular thickness (Tb.T), bone mineral density (BMD), trabecular separation (Tb.Sp), structure model index (SMI), cortical bone area (Ct.Ar) and cortical thickness (Ct.Th). **j** H&E staining of the sections from the distal femur. The data were confirmed by three repeated tests. The data are presented as the means ± SD. CA, catalpol. *P < 0.05 compared with the OVX group, ^#^*P* < 0.05 compared with the OVX + CA group

MicroCT scanning provided three-dimensional images of the microstructure of the distal femur (Fig. 6a). Compared with the OVX group, the OVX + CA group showed significantly enhanced BV/TV, Tb.N, BMD, Ct.Ar and Ct.Th (*P* < 0.05) and significantly decreased Tb.Sp and SMI (*P* < 0.05) (Fig. 6b–i). Consistent with the microCT analysis results, H&E staining of the distal femur sections showed that the number of trabeculae in the OVX + CA group was increased compared with that observed in the OVX group (Fig. 6j).

## Discussion

In the present study, by assessing calcium deposition and the expression of osteogenic-specific marker genes and proteins, we observed that catalpol could enhance the osteogenic differentiation of BMSCs. Subsequently, we showed that this phenomenon was accompanied by the upregulation of Wnt/β-catenin signalling. To confirm the involvement of the Wnt/β-catenin signalling cascade, we evaluated the inhibitory effect of this pathway and observed that DKK1, an antagonist of Wnt/β-catenin, could partially inhibit the increased osteogenesis of BMSCs due to catalpol treatment. Furthermore, to verify the effect of catalpol on bone regeneration, we utilized two widely used animal models, showing that catalpol could increase the bone healing capacity of BMSCs in a rat critical-sized calvarial defect model and attenuate bone loss in a rat ovariectomy model. These findings indicate that catalpol promotes the osteogenic differentiation of BMSCs, at least partly through activation of the Wnt/β-catenin signalling pathway.

Postnatal bone regeneration comprises two major ossification pathways, namely, endochondral ossification and intramembranous ossification, and BMSCs play a central role in both pathways [29, 30]. Osteoblasts, which are directly differentiated from BMSCs, are particularly important during the initial formation and maintenance of bone ossification as well as in fracture repair [31]. Osteoblasts can synthesize proteoglycans and type I collagen, which forms unmineralized osteoid, as well as other non-collagenous proteins, such as osteopontin and osteocalcin [32]. Subsequently, bone matrix mineralization occurs, which is characterized by the release of phosphates and calcium to form hydroxyapatite crystals. Thus, a growing number of researchers are working to identify methods that can differentiate BMSCs into osteoblasts to promote bone regeneration [29].

Increasing the osteogenic capacity of BMSCs is of great value for translational medicine. First, as promising seed cells, BMSCs are widely used to promote bone healing [33]. In clinical trials, autologous BMSC transplantation was reported to be a simple and safe method to promote bone repair in vertebra and in the femoral head [34, 35]. Thus, bioactive molecules that can create an optimal osteogenic microenvironment for BMSCs are promising for future clinical applications to accelerate bone formation [29]. In the present study, we observed that catalpol could significantly enhance the bone healing capacity of BMSCs in rat bone defects, showing potential to be used in bone tissue engineering for the treatment of large bone defects. Moreover, promoting the differentiation of BMSCs into mature osteoblasts is a rapidly progressing strategy for treating osteoporosis, which is known as anabolic treatment [1]. Despite their side effects, anabolic agents, such as recombinant human parathyroid hormone and its amino terminal fragment teriparatide, have been approved for the treatment of osteoporosis [36]. Many anabolic agents, such as the monoclonal sclerostin antibodies romosozumab and BPS804, have demonstrated efficacy in lowering the risk of fractures in postmenopausal women in clinical trials [36]. In the present study, we showed that catalpol could attenuate bone loss in ovariectomized rats, indicating its potential for treating osteoporosis.

Bone tissue engineering approaches include a combination of three building blocks: supporting scaffolds, growth factors and active cells to regenerate functional tissues [37]. In the present study, using a tissue engineering approach, we mixed BMSCs (cells) and catalpol (growth factors) into a bioactive hydrogel (scaffolds), and this system was demonstrated to be promising for regenerating bone. Recently, the pretreatment of seed cells with drugs/bioactive molecules for a period of time (several hours to a few days) was shown to be a potential strategy to amplify the therapeutic effect of MSCs after transplantation [38, 39]. A recent study by Lee et al. demonstrated that BMP-12 pretreatment of BMSCs for 12 h in vitro markedly increased the expression of tenocyte lineage markers over 14 days. The BMP-12-pretreated BMSCs were then seeded onto collagen scaffolds and implanted into an Achilles defect. After 21 days, the use of BMSCs pretreated with BMP-12 led to improved tendon repair compared with the untreated BMSCs [40]. Similarly, we observed that catalpol-pretreated BMSCs significantly enhanced bone regeneration in rat bone defects compared with the untreated BMSCs at 8 weeks after implantation. However, the basis for the persistent effects of the pretreatment strategy is unclear. One possible reason is that the direct cellular responses to these drugs/bioactive molecules are intrinsically long-lived, possibly reflecting changes towards a new state of differentiation [40].

Catalpol is primarily recognized as a neuroprotective agent for acute focal ischaemic stroke. Catalpol primarily exerts neuroprotective effects by reducing oxidative reactions, repressing inflammatory reactions and inhibiting apoptosis [12]. However, to the best of our knowledge, the effect of catalpol on the osteogenic differentiation of BMSCs has not been investigated. Catalpol has several obvious advantages as a potential therapy to enhance bone regeneration. First, catalpol is safe. This compound is enriched in and separated from the roots of Rehmanniae Radix, which is an official drug listed in the *Chinese Pharmacopoeia* and has been clinically used for more than 3000 years. In addition, the results of several studies have demonstrated that catalpol has no toxicity towards various cell types and no obvious adverse effects in animals [12, 41]. Second, catalpol is a convenient and stable compound, being soluble in water and easily transported and preserved. Third, catalpol is cheap and can be easily isolated from Rehmanniae Radix, which is planted in large quantities. The separation and purification processes have been well developed, and catalpol purity can reach more than 98% [41, 42]. Thus, catalpol may be a promising candidate for clinical trials.

In the present study, we observed that the inhibition of Wnt/β-catenin signalling could only partially block the increased osteogenesis of BMSCs, indicating the involvement of other pathways in catalpol activity. Furthermore, we demonstrated that the noncanonical Wnt, MAPK and BMP pathways, which are also important in bone regeneration, were also involved in promoting catalpol activity. Interestingly, complicated cross talk is known to occur between these pathways during the osteogenic differentiation of MSCs [31, 43]. For example, Wnt/β-catenin signalling may interact with noncanonical Wnt signalling through RhoA or sclerostin [44, 45]; BMP2 is reported to serve as a downstream target gene of Wnt/β-catenin signalling in osteoblasts [4]; Wnt3a strongly activates p38 MAPK, and this p38 MAPK activation regulates Wnt/β-catenin signalling by regulating GSK3β [46]; and the ligands BMP2 and BMP4 were reported to activate p38 and ERK MAPK [47]. However, to date, the cross talk between these pathways has not been completely elucidated. Thus, future studies should focus on gaining an understanding of the detailed mechanisms of action of catalpol.

The present study has several limitations. First, as mentioned above, the molecular mechanisms of the osteogenic effects of catalpol have not been fully elucidated. Second, the optimal drug doses and the timing of drug administration, which are important information for future translational studies, have not been well investigated. Thus, future studies should focus on understanding the detailed mechanisms of action of catalpol and investigating its optimal usage in large animal models.

## Conclusions

Our results showed that catalpol enhances the osteogenic differentiation of BMSCs, partly via activation of the Wnt/β-catenin pathway. The use of catalpol may provide a new strategy for bone tissue engineering and can be a potential agent for the treatment of postmenopausal osteoporosis.

## Additional files

Additional file 1: In situ SA-β-Gal assay. Catalpol had no effect on the senescence of BMSCs at concentrations of 10, 50 and 250 μM. (A) Representative microscopic images. (B) The percentage of SA-β-Gal-positive cells in each group. The data were confirmed by three repeated tests. The data are presented as the means ± SD. (TIF 2201 kb)

Additional file 2: The effect of catalpol on chondrogenesis, adipogenesis, and the MAPK and BMP signalling of BMSCs was evaluated by WB. The data were confirmed by three repeated tests. The data are presented as the means ± SD. *P < 0.05 compared with the control group, #*P* < 0.05 compared with the 10 μM catalpol treatment group, Δ*P* < 0.05 compared with the 50 μM catalpol treatment group. (TIF 1433 kb)

Additional file 3: The dose-response effect of DKK1 on osteogenesis in BMSCs treated with catalpol. (A-B) The expression levels of osteogenic-specific and Wnt/β-catenin signalling-related proteins were determined by WB. (C) Alizarin Red staining. (D) Calcium deposition was determined by an optical density analysis. The data were confirmed by three repeated tests. The data are presented as the means ± SD. CA, catalpol. β-catenin (T), total β-catenin. β-catenin (N), nuclear β-catenin. *P < 0.05 compared with the control group, #*P* < 0.05 compared with the catalpol treatment group, Δ*P* < 0.05 compared with the group treated with catalpol + 0.1 μg/ml DKK1. (TIF 2199 kb)

Additional file 4 The involvement of the noncanonical Wnt pathway in the activity of catalpol. BMSCs were cultured in OIM supplemented with 50 μM catalpol in the presence or absence of 2 μM SP600125 or 0.1 μg/ml DKK. The expression levels of COL1 and RUNX2 were evaluated by WB after 7 days of osteogenic induction. The data were confirmed by three repeated tests. The data are presented as the means ± SD. CA, catalpol. *P < 0.05 compared with the control group, #*P* < 0.05 compared with the catalpol treatment group, Δ*P* < 0.05 compared with the catalpol + DKK1 treatment group. (TIF 760 kb)

Additional file 5 The inhibitory effect of DKK1 in a rat critical-sized calvarial defect model. Fifteen rats were randomly assigned to the following three implant groups: (1) hydrogel mixed with BMSCs (BMSCs group) (*n* = 5); (2) hydrogel mixed with BMSCs treated with catalpol (BMSCs + CA group) (*n* = 5); and (3) hydrogel mixed with BMSCs treated with catalpol + DKK1 (BMSCs + CA + DKK1 group) (*n* = 5). Four weeks after surgery, bone regeneration was evaluated. (A) MicroCT 3D reconstruction and coronal images of the defect area. (B-C) MicroCT analyses of the bone volume/total volume (BV/TV) and bone mineral density (BMD). The data are presented as the means ± SD. CA, catalpol. *P < 0.05 compared with the BMSCs group, #*P* < 0.05 compared with the BMSCs + CA group. (TIF 1759 kb)

## Abbreviations

ALP: Alkaline phosphatase
BMD: Bone mineral density
BMSCs: Bone marrow mesenchymal stem cells
BV/TV: The new bone volume/total volume
CA: Catalpol
CCK8: Cell Counting Kit-8
DKK1: Dickkopf-related protein 1
FBS: Foetal bovine serum
H&E: Haematoxylin and eosin
IF: Immunofluorescence
MB: Mineralized bone
OB: Osteoblasts
OIM: Osteogenic induction medium
OT: Osteocytes
OVX: Ovariectomized
PBS: Phosphate-buffered saline
P-GSK-3β: Ser9 phosphorylation GSK-3β
qRT-PCR: Quantitative reverse transcription polymerase chain reaction
RIPA: Radioimmunoprecipitation assay
SD: Standard deviation
Tb.N: Trabecular number
Tb.T: Trabecular thickness
UB: Unmineralized bone
VG: van Gieson’s
WB: Western blot
α-MEM: Modified Eagle’s medium alpha modification
