# Supplementary figures and images for: Catalpol promotes the osteogenic differentiation of bone marrow mesenchymal stem cells via the Wnt/β-catenin pathway
Source: Stem Cell Res Ther. 2019 Jan 22;10:37. doi: 10.1186/s13287-019-1143-y (PMC6341609; doi:10.1186/s13287-019-1143-y)

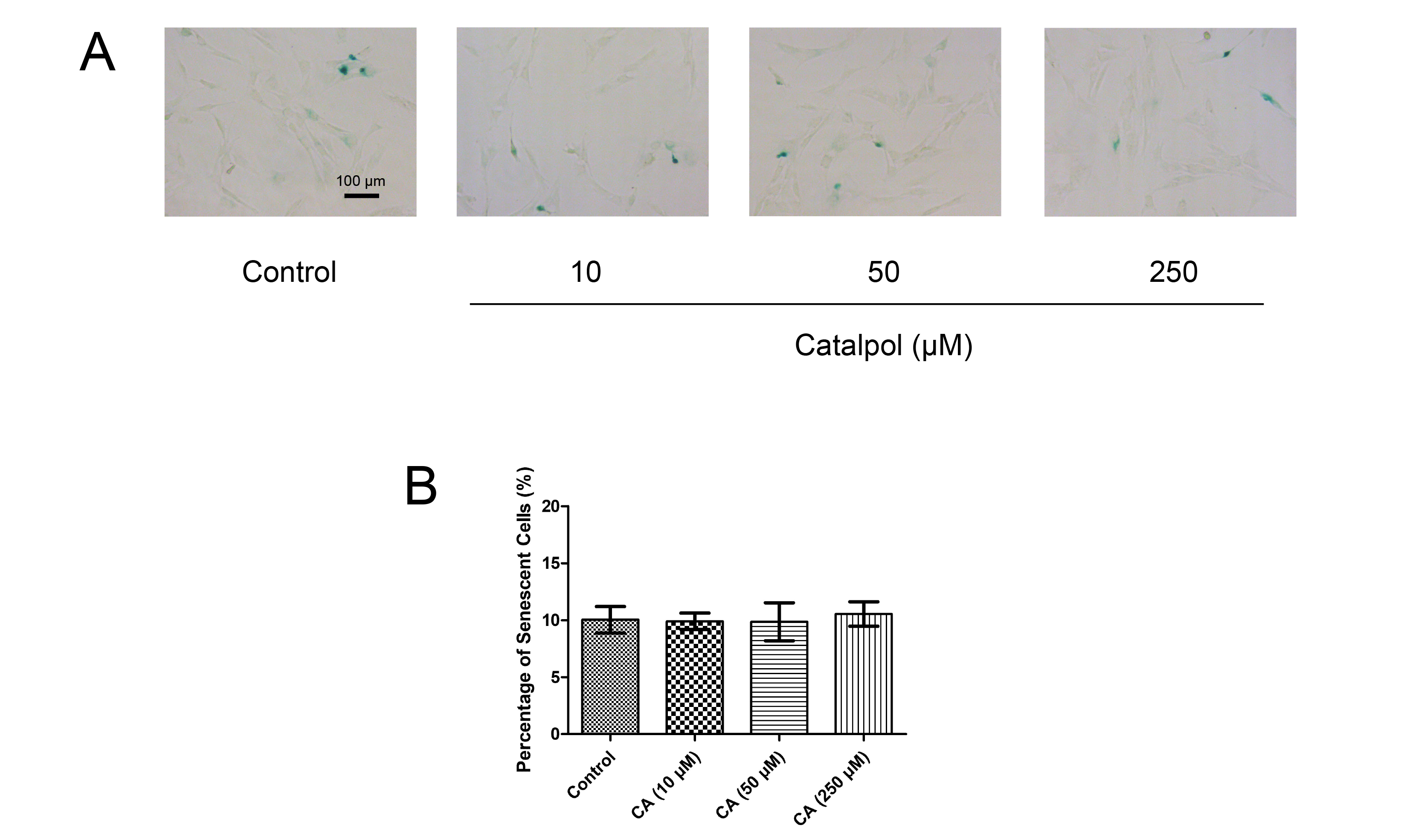

Supplement: Supplementary file 1 — In situ SA-β-Gal assay. Catalpol had no effect on the senescence of BMSCs at concentrations of 10, 50 and 250 μM. (A) Representative microscopic images. (B) The percentage of SA-β-Gal-positive cells in each group. The data were confirmed by three repeated tests. The data are presented as the means ± SD. (TIF 2201 kb) [file 13287_2019_1143_MOESM1_ESM.tif]

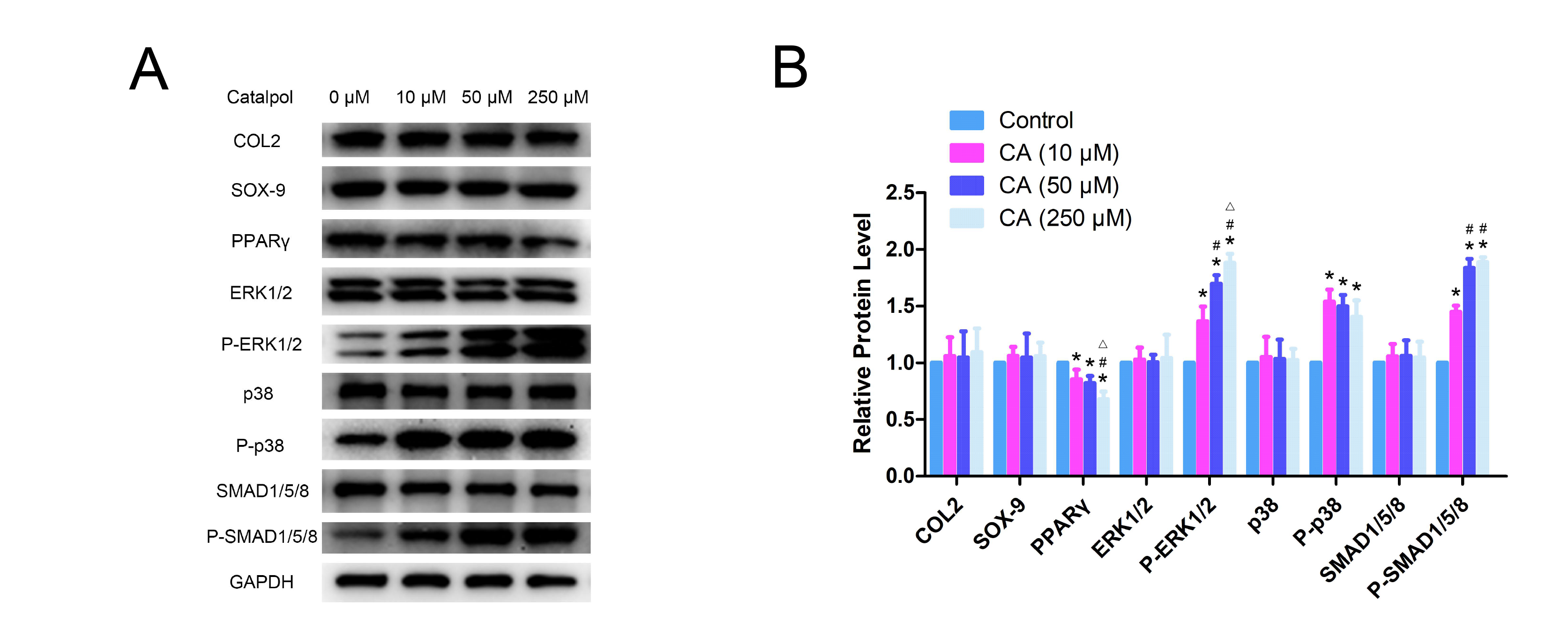

Supplement: Supplementary file 2 — The effect of catalpol on chondrogenesis, adipogenesis, and the MAPK and BMP signalling of BMSCs was evaluated by WB. The data were confirmed by three repeated tests. The data are presented as the means ± SD. *P < 0.05 compared with the control group, #P < 0.05 compared with the 10 μM catalpol treatment group, ΔP < 0.05 compared with the 50 μM catalpol treatment group. (TIF 1433 kb) [file 13287_2019_1143_MOESM2_ESM.tif]

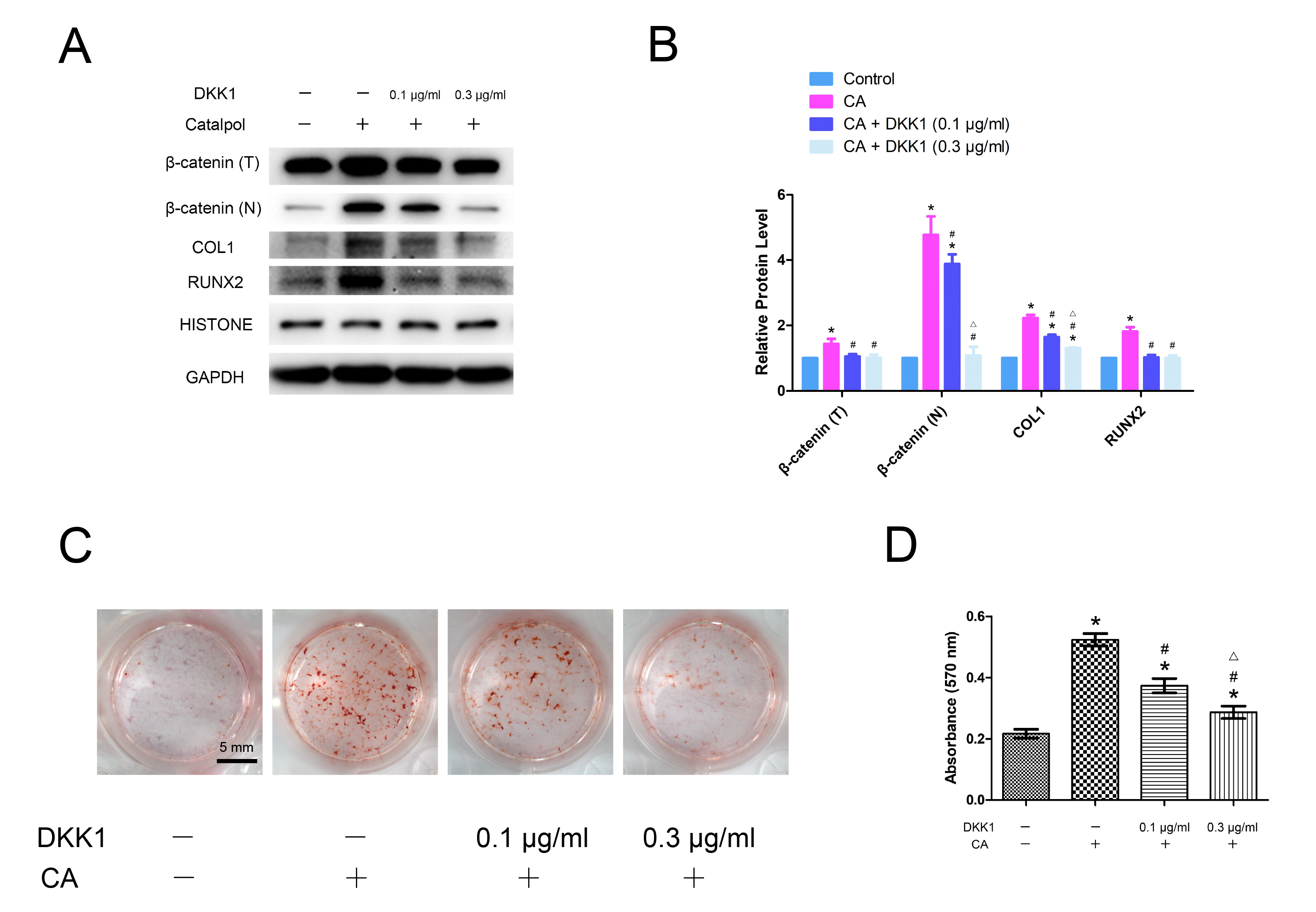

Supplement: Supplementary file 3 — The dose-response effect of DKK1 on osteogenesis in BMSCs treated with catalpol. (A-B) The expression levels of osteogenic-specific and Wnt/β-catenin signalling-related proteins were determined by WB. (C) Alizarin Red staining. (D) Calcium deposition was determined by an optical density analysis. The data were confirmed by three repeated tests. The data are presented as the means ± SD. CA, catalpol. β-catenin (T), total β-catenin. β-catenin (N), nuclear β-catenin. *P < 0.05 compared with the control group, #P < 0.05 compared with the catalpol treatment group, ΔP < 0.05 compared with the group treated with catalpol + 0.1 μg/ml DKK1. (TIF 2199 kb) [file 13287_2019_1143_MOESM3_ESM.tif]

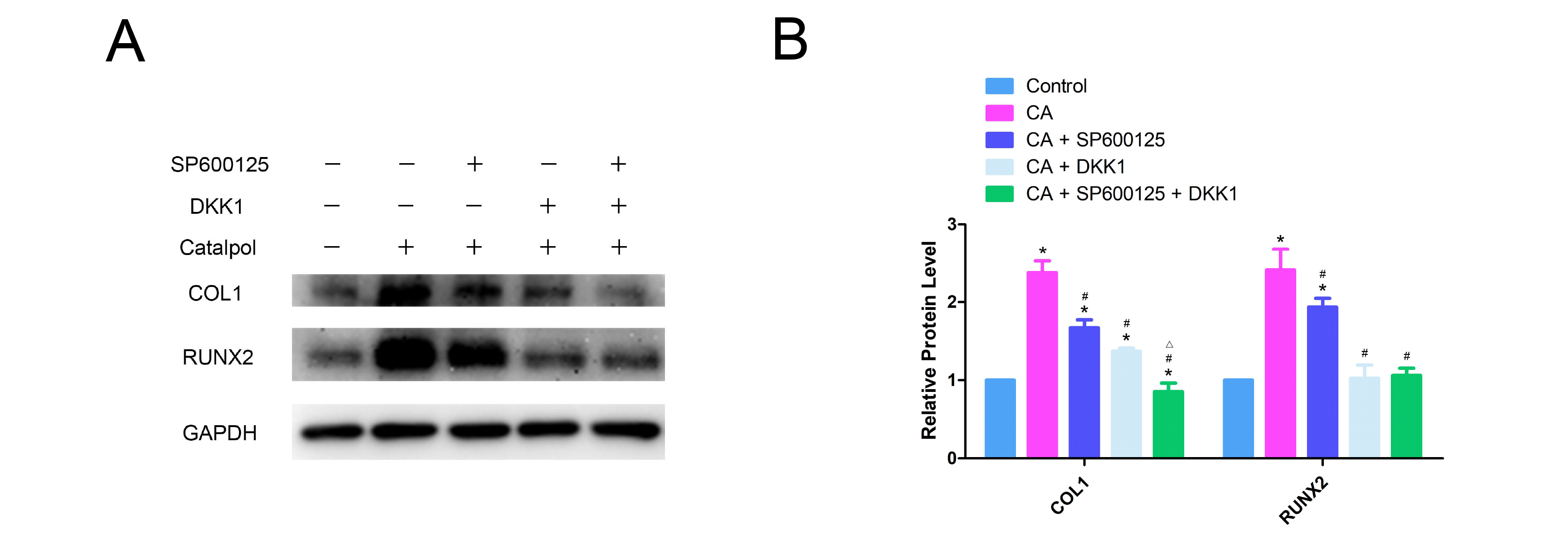

Supplement: Supplementary file 4 — The involvement of the noncanonical Wnt pathway in the activity of catalpol. BMSCs were cultured in OIM supplemented with 50 μM catalpol in the presence or absence of 2 μM SP600125 or 0.1 μg/ml DKK. The expression levels of COL1 and RUNX2 were evaluated by WB after 7 days of osteogenic induction. The data were confirmed by three repeated tests. The data are presented as the means ± SD. CA, catalpol. *P < 0.05 compared with the control group, #P < 0.05 compared with the catalpol treatment group, ΔP < 0.05 compared with the catalpol + DKK1 treatment group. (TIF 760 kb) [file 13287_2019_1143_MOESM4_ESM.tif]

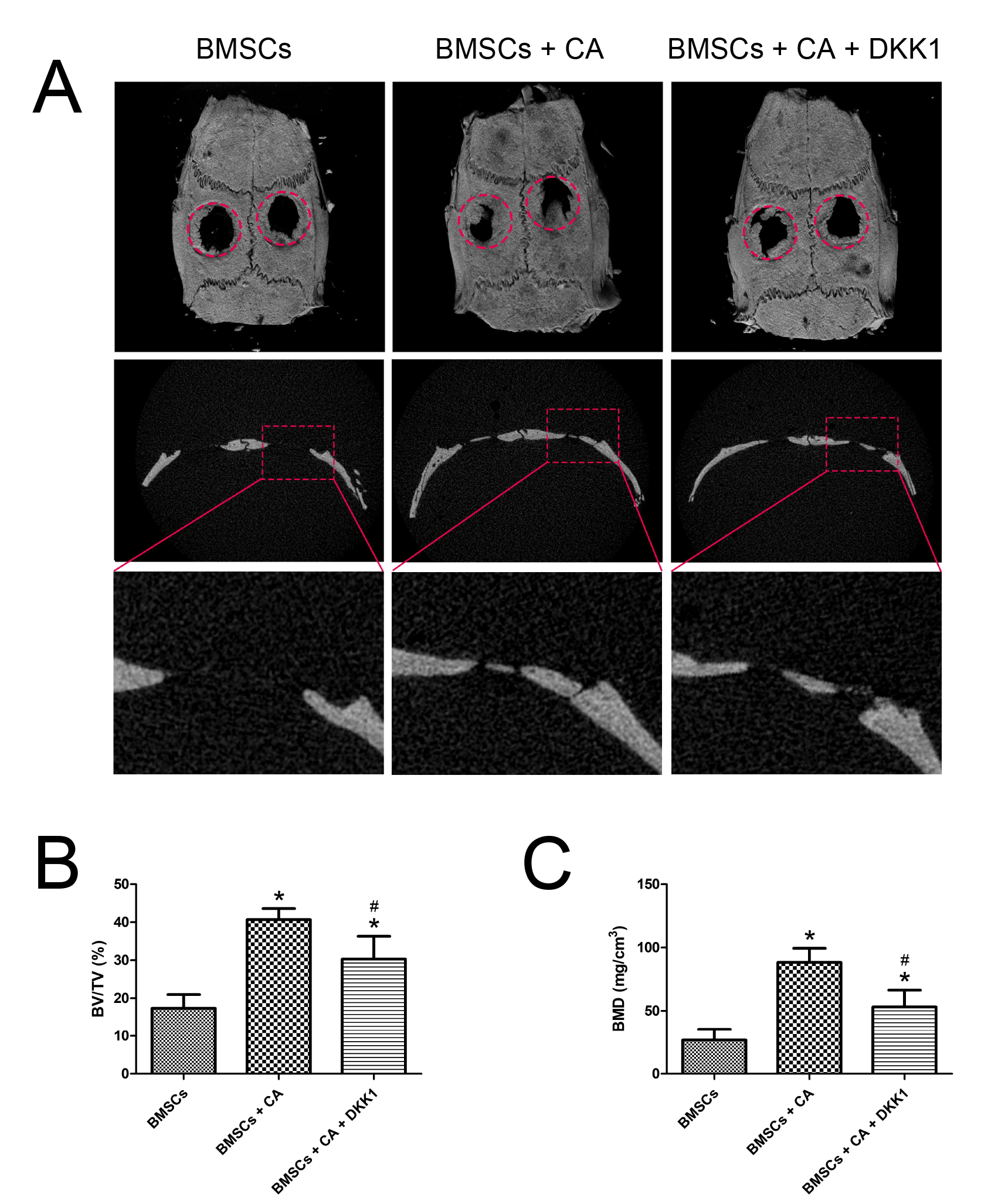

Supplement: Supplementary file 5 — The inhibitory effect of DKK1 in a rat critical-sized calvarial defect model. Fifteen rats were randomly assigned to the following three implant groups: (1) hydrogel mixed with BMSCs (BMSCs group) (n = 5); (2) hydrogel mixed with BMSCs treated with catalpol (BMSCs + CA group) (n = 5); and (3) hydrogel mixed with BMSCs treated with catalpol + DKK1 (BMSCs + CA + DKK1 group) (n = 5). Four weeks after surgery, bone regeneration was evaluated. (A) MicroCT 3D reconstruction and coronal images of the defect area. (B-C) MicroCT analyses of the bone volume/total volume (BV/TV) and bone mineral density (BMD). The data are presented as the means ± SD. CA, catalpol. *P < 0.05 compared with the BMSCs group, #P < 0.05 compared with the BMSCs + CA group. (TIF 1759 kb) [file 13287_2019_1143_MOESM5_ESM.tif]
